# Study on efficacy and safety of Tong-luo Qu-tong plaster treatment for knee osteoarthritis: study protocol for a randomized, double-blind, parallel positive controlled, multi-center clinical trial

**DOI:** 10.1186/s13063-019-3481-6

**Published:** 2019-06-24

**Authors:** Bao-ping Xu, Min Yao, Zi-rui Tian, Long-yun Zhou, Long Yang, Zhen-jun Li, Sen Zhu, Xiao-tao Wang, Jia-hui Lan, Yong-jun Wang, Xue-jun Cui

**Affiliations:** 1 Spine Disease Institute, Longhua Hospital, Shanghai University of Traditional Chinese Medicine, 725 South Wanping Road, Shanghai, 200032 China; 2 Key Laboratory of Theory and Therapy of Muscles and Bones, Ministry of Education (Shanghai University of Traditional Chinese Medicine), 725 South Wanping Road, Shanghai, 200032 China; 3 Lu’an Hospital of Traditional Chinese Medicine, 76 Renmin Road, Anhui, 237000 Lu’an China; 4 Shanghai Guanghua Hospital of Integrated Traditional Chinese and Western Medicine, 540 Xinhua Road, Shanghai, 200052 China; 5 Gansu Provincial Hospital of Traditional Chinese Medicine, 418 Guazhou Road, Qi lihe District, Gansu, 730050 Lanzhou China; 6 Department of Orthopaedic, Shanghai Pudong Gongli Hospital, Second Military Medical University, 219 Miaopu Road, Shanghai, 200013 China; 7 Ehu Branch of Xishan People’s Hospital, No. 1 Xuehai East Road, Xishan District, Wuxi, 214116 Jiangsu China; 8 Longhua Hospital, Shanghai University of Traditional Chinese Medicine, 725 South Wanping Road, Shanghai, 200032 China

**Keywords:** Clinical trials, Knee osteoarthritis, Tong-luo Qu-tong plaster, Randomized, Protocol

## Abstract

**Background:**

Knee osteoarthritis (KOA) is a common chronic musculoskeletal disorder that seriously affects quality of life. Patients with KOA frequently develop one or more of the following typical symptoms: joint pain, stiffness, joint friction noise and impaired functionality. Traditional Chinese medicine (TCM) has been shown to have a superior effect and a particular advantage in the treatment of KOA; among TCM, the Tong-luo Qu-tong plaster is the convenient and most commonly used method in China to improve symptoms including pain, stiffness and limited mobility in patients with KOA, as it causes few adverse effects. But there is a lack of high-quality clinical evidences to support the therapeutic effect that Chinese adhesive plaster can have in relieving pain and stiffness. The purpose of this study will be to evaluate the efficacy and safety of Tong-luo Qu-tong plaster in patients with KOA.

**Methods/design:**

This study will be a randomized, double-blind, parallel positive controlled, multi-center clinical trial, a non-inferiority trial design was adopted. A total of 2000 participants older than 40 years, with KOA, will be randomly allocated into an experimental group (*n* = 1500) and a control group (*n* = 500). All participants will receive a conventional conservative treatment lasting for 14 days as two courses, once daily. Tong-luo Qu-tong plaster will be administered externally to participants in the experimental group, while the control group will receive a Qi-zheng Xiao-tong plaster. The outcome of the total Western Ontario and McMaster Universities Arthritis Index scores, TCM syndrome quantitative score and visual analog scale scores will be measured during the assessment visits (baseline and 1-week and 2-week follow up). In addition, adverse events related to clinical symptoms and signs and results of laboratory tests will be documented during the clinical trials.

**Discussion:**

This study will provide reliable evidence of the effectiveness and safety of Tong-luo Qutong plaster in patients with KOA. If the results are favorable, it is expected that the patients with KOA will benefit from this study, many patients may have a good alternative treatment for KOA.

**Trial registration:**

ClinicalTrials.gov, ID: NCT03309501. Registered on 8 November 2017.

**Electronic supplementary material:**

The online version of this article (10.1186/s13063-019-3481-6) contains supplementary material, which is available to authorized users.

## Background

Knee osteoarthritis (KOA), also known as degenerative arthritis, is a kind of chronic joint disease characterized by the progressive degeneration and breakdown of the articular cartilage and bone hyperplasia [1]. Patients with severe osteoarthritis frequently develop one or more of the following typical symptoms: joint pain, stiffness, joint friction noise on climbing stairs and impaired functionality such as difficulty in walking and climbing [2, 3]. It has been estimated that the worldwide prevalence of symptomatic osteoarthritis (OA) is more than 10% in people above 60 years old, and in an epidemiological survey the overall prevalence of OA in a rural Chinese adult population was about 16% [4]. KOA affects over 70 million people in Europe and the direct medical costs exceed 2 billion Euros, which represents a social, economic burden and KOA was the 11th leading cause of disability according to the World Health Organization (WHO) global burden of disease study 2010 [1, 5]. Patients with KOA are often treated surgically and managed conservatively [6]; surgical treatment includes total knee arthroplasty (TKA), arthroscopic surgery and so on and is well-known to substantially reduce KOA-related pain and improve function [7, 8]. However, some patients are not actually willing to undergo TKA, due in large part to lack of confidence in beneficial surgery outcomes and postoperative complications such as chronic pain after TKA, which can affect all dimensions of health-related quality of life [9, 10]. Additionally, each of these surgical options permanently modifies the knee joint via an invasive, irreversible surgical procedure, which may also negatively impact patient willingness to undergo these procedures and limits clinical utility. Therefore, conservative treatment such as non-steroidal anti-inflammatory drugs (NSAIDs), paregoric, cartilage-protective agents and so on play an important role in the treatment of KOA.

However, the application of NSAIDs is reported to lead to some adverse effects, including gastrointestinal tract impairment and possible promotion of articular deterioration [6, 11]. Cartilage protective agents such as glucosamine and chondroitin have caused troubles and concerns for clinicians due to lack of effectiveness over recent decades [12]. The efficacy of glucosamine has been questioned in a randomized, controlled, double-blind, placebo study, as there was no significant clinical benefit compared to the placebo group [13]. Traditional Chinese medicine (TCM) has been shown more effective and has a unique advantage in the treatment of KOA, as the herbal plaster is a common approach and a convenient choice for many patients with KOA [14]. Our earlier studies also proved the safety of Tong-luo Qu-tong plaster, which can help significantly reduce pain and improve function with better clinical curative effect and no serious adverse reactions in people with KOA [15, 16]. But with the lack of a large-sample, randomized, double-blind, controlled clinical trial, further clinical evidence on Tong-luo Qu-tong plaster in the treatment of KOA is needed. Therefore, the purpose of this study is to assess the effectiveness and safety of Tong-luo Qu-tong plaster in patients with KOA in a randomized, double-blind, parallel positive controlled, multicenter clinical trial.

## Methods and designs

### Trial design

This will be a randomized, double-blind, parallel positive controlled, multicenter clinical trial; a non-inferiority trial design was adopted. With the unceasing development of medical technology, there are increasing numbers of positive drugs with the exact curative effect in clinical treatments. Once a treatment has been established as effective, it would be unethical to undertake placebo-controlled trials [17], which has led to more widespread application of clinical non-inferiority trials over recent decades [18, 19]. A non-inferiority trial design could be a better alternative to indirectly show the efficacy of a new treatment [20].

Each participant will sign an informed consent form (ICF) before the research is performed. A total of 11 medical institutions are involved in the study; subjects will be enrolled at eleven hospitals, including Longhua Hospital Affiliated to Shanghai University of TCM, The first Affiliated hospital of Guangzhou University of TCM, Zhengzhou Central Hospital, Suzhou Hospital of TCM, Luoyang Orthopaedics-Traumatological Hospital, Xiangyang First People’s Hospital, Liaoning Hospital of TCM, The Second Hospital of Nanjing Medical University, The Fourth Central Hospital of Tianjing, Changchun University of TCM and Shandong University of TCM. Longhua Hospital Affiliated to Shanghai University of TCM takes charge of the total clinical scheme design. The study protocol has been approved by the ethics committee of Shanghai University of TCM on the use of human subjects for research (approval number 2016LCSY097); each participating center has conducted ethical filing and has ethical approval from the main central hospital.

**Fig. 1:**
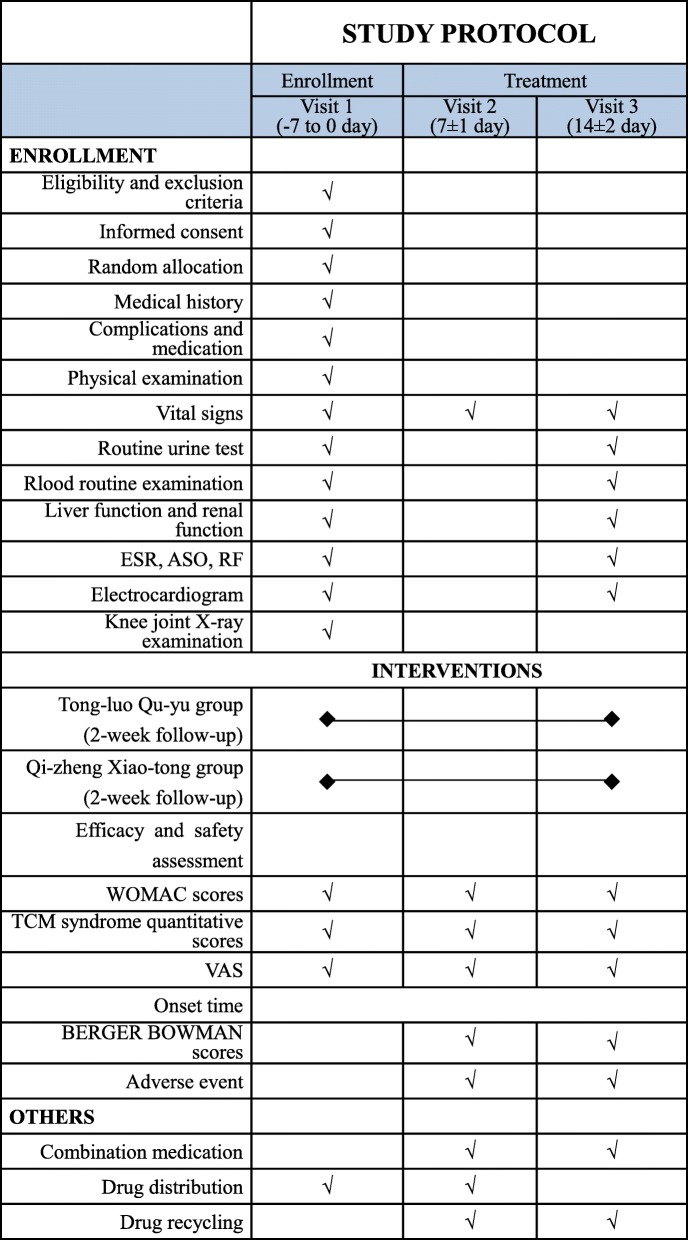
Study phases schedule of the randomized controlled trial for patients

**Fig. 2:**
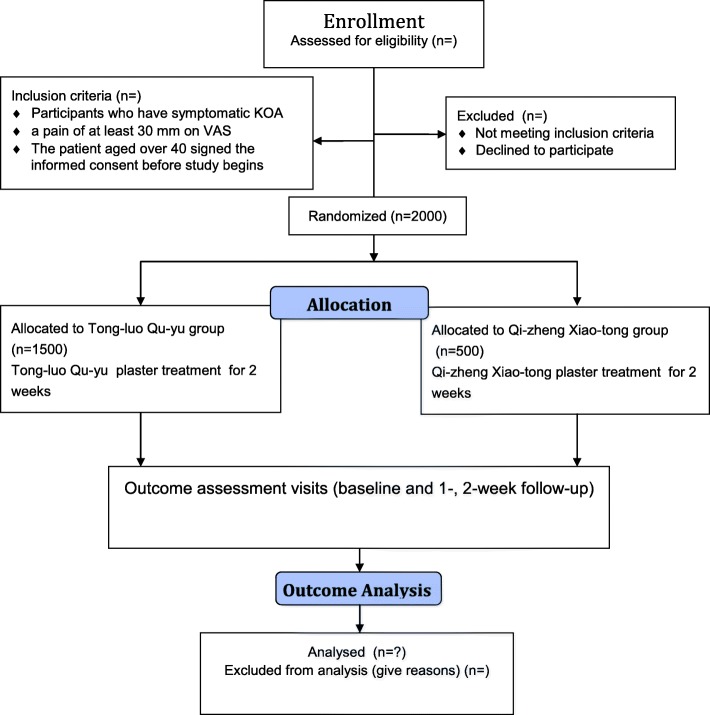
Study flow diagram of trial participation

The study phases are shown in Fig. 1. A total of 2000 patients with KOA will be recruited and randomly allocated into the experimental group (*n* = 1500) or the control group (*n* = 500); each patient will undergo a 2-week treatment with herbal patches for one session per day. A flow chart of trial participation is provided in Fig. 2. Efficacy and safety data will be collected throughout the whole study.

### Inclusion criteria

The following inclusion criteria should be met:

1. Participants with symptomatic KOA, with diagnosis based on criteria developed by the American College of Rheumatology (ACR) in 1986 [21]
2. Standard TCM disease and syndrome diagnosis [22, 23]
3. Symptomatic KOA with a pain score of at least 30 mm on a 100-mm visual analog scale (VAS)
4. Age ≤ 20 years
5. Must have signed the ICF before the study begins

Moreover, if the patient has osteoarthritis in both knees, we will choose the more severe side of the knee joint. If the pain scores are the same in both knees, the researchers will choose one side of the knee joint for intervention according to the research demands.

### Exclusion criteria

The exclusion criteria are:

1. History of trauma or surgery at the knee joint in the last 6 months before the trial begins
2. Arthroscopy and intra-articular injection performed in the last 3 months before the trial begins, hormone therapy in the first month of screening or knee arthroplasty
3. Current participation or participation within the last 3 months in other clinical trials
4. Other knee joint diseases such as chondromalacia patellae, rheumatic arthritis or rheumatoid factor (RF)-positive (RF > 40 U/ml);
5. Mental disorder or severe diseases and complications such as severe diabetes mellitus, serious liver and kidney disease, malignant tumors, infectious diseases or complications affecting the joints
6. Plaster allergy or pregnant or lactating

### Intervention

All patients will be randomly divided into either the Tong-luo Qu-tong plaster group (experimental group) or the Qi-zheng Xiao-tong plaster (control group), the experimental group and the control group will receive Tong-luo Qu-tong plaster or Qi-zheng Xiao-tong plaster, respectively. Tong-luo Qu-tong plaster is a tape-type Chinese herbal patch, composed of *Syzygium aromaticum*, *Zanthoxylum bungeanum*, cinnamon, *Rhizoma zingiberis*, borneol, camphor, menthol crystal and a hydrophilic adhesive vehicle. It is made by Henan Lingrui Pharmaceutical Ltd. (State Food and Drug Administration approval number Z20000065); the validity period of Tong-luo Qu-tong plaster is 24 months. Qi-zheng Xiao-tong plaster is a medicated plaster made by Tibet Qizheng Tibetan Medicine Ltd. (State Food and Drug Administration approval number Z54020113); it is valid for 36 months. The main components of the Qi-zheng Xiao-tong plaster are *Lamiophlomis rotate*, *Curcuma longa* and a hydrophilic adhesive vehicle. The components of two applications in this trial differ, but the two kinds of plaster are identical in terms of texture, size, color and odor. Both groups use the plaster according to the instructions given by nurses, and all participants will receive a conventional conservative treatment as two courses over 14 days, once daily. Patients will have three follow-up visits; the clinicians, subjects, investigators and assessors will be masked to treatment allocation. In the process of trial, patients are not allowed to use other types of TCM. Subjects in severe pain (with VAS scores > 80 mm) may be given celebrex to relieve pain in two daily doses; If patients have other accompanying diseases that require treatment, the necessary interventions are permissible, but only if they do not affect the evaluation of this clinical trial.

### Safety assessment

Adverse events (AEs) related to clinical symptoms and signs and the results of laboratory tests will be documented during the clinical trial. Skin irritation will be recorded using the Berger Bowman scoring system [24], and subjective symptoms including itching, pain, burning sensation and skin lesions manifesting as erythema, papules, edema, blisters, erosions, skin ulcers and so on will be recorded after 1 and 2 weeks of treatment. Drug safety will be monitored by blood routine examination (BRE), urine routine test (URT), liver function tests (LFTs) including measurement of serum glutamic oxaloacetic transaminase (AST), serum glutamic pyruvic transaminase (ALT), serum total protein (TP), serum alkaline phosphatase (ALP) and serum total bilirubin (TBIL), kidney function tests (KFT) including measurement of blood urea nitrogen (BUN) and serum creatinine, between the start and end of the trial. Erythrocyte sedimentation rate (ESR), anti-streptococcus hemolysin (ASO) and rheumatoid factor (RF) will also be recorded and electrocardiogram (ECG) and x-ray examination will be performed. Serious adverse events (SAEs) will be reported to the local drug administration authorities within 24 h.

### Outcome assessment

Outcome assessment are based on the *Guidelines for clinical research on Chinese new herbal medicines* and *Standards for diagnosis and curative effect of Chinese medical symptom* [22, 23].

#### Primary outcome

The Western Ontario and McMaster Universities Osteoarthritis Index (WOMAC) as an objective indicator of efficacy is the primary efficacy endpoint of the study; this is a widely used, proprietary set of standardized questionnaires used by health professionals to evaluate the condition of patients with osteoarthritis of the knee and hip, including pain, stiffness, and physical functioning of the joints [25]. The WOMAC measures 5 items for pain (score range 0–20), 2 for stiffness (score range 0–8) and 17 for functional limitation (score range 0–68) to assess the severity of arthritis and the therapeutic effect according to the patients’ symptoms and signs. It can fully reflect the basic situation of osteoarthritis [26]. The primary outcome is improvement in total WOMAC scores, which will be measured during the assessment visits (baseline and 1-week and 2-week follow up).

#### Secondary outcome assessment

The secondary outcome is change between baseline and the end of treatment in the TCM syndrome quantitative score [23], VAS scores [27] and time of onset of pain relief after administration of the drug. The VAS scores range from 0 mm to 100 mm, and it is widely used for clinical evaluation of the degree of pain. The time when the VAS score was reduced by at least 10 mm for the first time after administration of the drug was recorded, namely the time of onset of pain relief.

### Sample size estimation

Our study was designed as a non-inferiority trial and sample size calculations are based on the primary outcome measurement. First, the minimal clinically important difference in WOMAC scales in KOA is estimated from previous studies [28] as 15.50 points. Second, we assume that based on previous literature [29], the square deviation of the WOMAC score is 318.88. For power of 80% and an alpha value of 2.5% (two-tailed), the sample size is calculated using the following formula:

$$ n=\frac{\frac{4}{3}{\left({u}_{\upalpha}+{u}_{\beta}\right)}^2{\upsigma}^2}{{\left(\varDelta -\updelta \right)}^2}\ \left({u}_{\upalpha}=1.6449,\kern0.5em {u}_{\beta }=1.2816\right) $$

Thus, we obtained the sample size of 1600 patients for this trial; allowing for a conservative 20% dropout rate, the total sample size was set at 2000 patients (1500 in the Tong-luo Qu-tong plaster group).

### Randomization

This study is designed as a randomized, double-blind, parallel positive drug controlled, multicenter clinical trial. A total of 2000 eligible participants will be randomized (3:1) using a stratified-block randomization method based on the disease and the center to two treatment groups: the experimental group (Tong-luo Qu-tong group) and the control group (Qi-zheng Xiaotong group). A number of previous clinical studies also had adopted unequal allocation-ratio designs, which allows the minimization of potentially unethical exposure of patients to a placebo [30–32]. However, our study lacked a placebo group, which is different from previous studies. Based on the above, we drew up an appropriate unequal allocation ratio. All eligible patients were assigned in a 3:1 ratio (Tong-luo Qu-tong plaster group: Qi-zheng Xiao-tong plaster group) by a stratified-block randomization method; the study design ensured a greater number of patients undergoing Tong-luo Qu-tong plaster exposure by employing an unequal allocation ratio. This provided better safety assessment and greater exposure for testing the efficacy of Tong-luo Qu-tong plaster in this randomized controlled clinical trial, to generate good evidence to answer the trial research question. SAS statistical software 9.2 (SAS Institute, Cary, NC, USA) will be used to generate a randomization scheme based on the PROC PLAN function, which will be used to link the patient to a treatment arm and will specify a unique medication number for the first package of study drug to be dispensed to the patient.

### Drug management

In this clinical trial, one experimental drug administrator was assigned to perform drug management including drug storage, distribution, and recycling independently and kept detailed records. The whole process of drug coding and documentation is performed blinded. The drug boxes and emergency envelope contain the corresponding drug numbers, which are randomly divided between each center according to the central number for random stratification. Emergency unblinding can only happen when there a serious adverse event occurs in the study; in this event, the subject will exit the trial and the investigator will record detailed reasons for the subject’s withdrawal from the trial in the case report form.

### Statistical analysis

The statisticians and the main researchers are responsible for the statistical analysis plan. We will analyze all data with SAS 9.2 statistical software. Data sets including the full analysis set (FAS), per-protocol set (PPS) and safety set (SS), will be analyzed in terms of actual subjects, shedding cases, excluding cases, demographic and characteristics of cases, and efficacy and safety analyses will be conducted according to the intention-to-treat (ITT) principle.

Categorical data presented as frequency tables or percentages and continuous data presented as mean ± standard deviation, median, superior and inferior quartiles, minimum value and maximum value will be used to describe the characteristics of patients in both groups. The primary outcome will be compared in the two groups; categorical data will be analyzed by chi-squared test or Fisher’s exact test, and continuous data with a normal distribution will be analyzed by the *t* test or variance testing. If the data are not normally distributed or do not satisfy homogeneity of variance, they will be analyzed using the Wilcoxon rank sum test or Wilcoxon symbols test to compare the two treatment arms. A two-sided *P* value ≤0.05 or ≤0.01 will be considered statistically significant.

## Discussion

KOA is a common degenerative disease, particularly in older adults. In the last 20 years, there was an increase of about 26% in the burden of KOA as measured by years lived with disability per 100,000 persons [33]. Tong-luo Qu-tong plaster, also known as external medication, is a conventional method of treating chronic musculoskeletal diseases in TCM. The treatment is convenient and inexpensive [15, 16] but there is a lack of high-quality clinical evidence to support the claimed therapeutic effect of Chinese adhesive plaster in relieving pain and stiffness. Well-designed, randomized controlled trials are needed to examine the efficacy of TCM treatments for KOA, and the objective of this clinical trial is to evaluate the efficacy and safety of Tong-luo Qu-tong plaster in patients with KOA. The study is guided by practice-based scientific evidence for the use of Tong-luo Qu-tong plaster for this condition. Upon completion of data collection, it is expected that the patients with KOA will benefit from this study. The data will be published after the study is completed.

The study is designed in accordance with Standard Protocol Items: Recommendations for Interventional Trials (SPIRIT) guidelines. The SPIRIT checklist is given in Additional file 1.

### Trial status

The trial was registered at ClinicalTrials.gov on 8 November 2017 (identifier NCT03309501), and protocol version 2.1/20170923 is currently active. We started recruitment in September 2017 and it will be completed in December 2020. The first patient to be included was at the Second Hospital of Nanjing Medical University.

## Additional file

Additional file 1: SPIRIT 2013 checklist: Recommended items to address in a clinical trial protocol and related documents*. (DOC 136 kb)

## Data Availability

The full data have not yet been collected in this study and therefore are not yet available to the public. Trial information can be found at ClinicalTrials.gov, NCT0330950.

## Abbreviations

ACR: American College of Rheumatology
AE: Adverse event
ALP: Serum alkaline phosphatase
ALT: Serum glutamic pyruvic transaminase
ASO: Anti-streptococcus hemolysin
AST: Transaminase
BRE: Blood routine examination
BUN: Blood urea nitrogen
ECG: Electrocardiography
ESR: Erythrocyte sedimentation rate
FAS: Full analysis set
ICF: Informed consent form
ITT: Intention-to-treat
KFT: Kidney function tests
KOA: Knee osteoarthritis
LFT: Liver function test
NSAID: Non-steroidal anti-inflammatory drug
PPS: Per-protocol set
RF: Rheumatoid factor
SAE: Serious adverse event
SPIRIT: Standard Protocol Items: Recommendations for Interventional Trials
SS: Safety set
TBIL: Serum total bilirubin
TCM: Traditional Chinese medicine
TP: Serum total protein
URT: Urine routine test
VAS: Visual analog scale
WHO: World Health Organization
WOMAC: Western Ontario and McMaster Universities Arthritis Index scores
